# Kinase Regulation Through Metabolic Control Points Directs Glucose Fate

**DOI:** 10.3390/ijms27219402

**Published:** 2026-10-22

**Authors:** Susmi Varghese, Prathik Basthikoppa Shivamurthy, Vinitha Ramanath Pai, Sowmya Soman, Poornima Ramesh, Rajesh Raju

**Affiliations:** 1 Centre for Integrative Omics Data Science (CIODS), Yenepoya (Deemed to be University), Mangalore 575018, Karnataka, India; elizabethsusmi@gmail.com (S.V.); prathikbsgowda@gmail.com (P.B.S.); sowmyasoman.ciods@yenepoya.edu.in (S.S.); poornima_r.ciods@yenepoya.edu.in (P.R.); 2 Centre for Systems Biology and Molecular Medicine (CSBMM), Yenepoya Research Centre, Yenepoya (Deemed to be University), Mangalore 575018, Karnataka, India; 3 Department of Biochemistry, Yenepoya Medical College, Yenepoya (Deemed to be University), Mangalore 575018, Karnataka, India; vinitharpai@gmail.com

**Keywords:** glucose metabolism, kinase–metabolic enzyme associations, phosphoproteomics, metabolic control points, protein–protein interaction network, systems biology

## Abstract

Glucose metabolism is organized through interconnected metabolic pathways whose enzymes determine whether glucose is utilized for energy production, storage, or biosynthesis. Although protein kinases modulate metabolic enzymes through phosphorylation, how kinase signaling collectively interfaces with the glucose metabolic network remains unclear. In addition, many reported kinase–substrate relationships are derived from prediction-based resources and lack experimental validation. Here, we systematically mapped kinase associations for 144 enzymes across six glucose metabolic pathways by integrating kinase–substrate databases, quantitative phosphoproteomics from 154 human studies, and protein–protein interaction networks. We developed a multi-layer prioritization framework combining kinase–substrate annotations, phosphoproteomic co-regulation, and interaction-supported evidence to rank kinase–enzyme associations and reconstruct a phosphoproteomic-supported kinase–metabolism network. Prioritization reduced a large initial association space to a refined set of candidate kinase–enzyme associations while retaining broad coverage across glucose metabolism. The resulting network revealed an uneven distribution of kinase associations across metabolic pathways and enzymes, with the highest kinase association density observed at metabolic control points, particularly enzymes integrating branch-point and flux-control functions. Pathway-level analysis further showed that glycogen metabolism exhibited the greatest kinase association density despite comprising relatively few enzymes, while futile-cycle pairs displayed distinct patterns of shared and reaction-specific kinase associations. Network analysis identified a small subset of highly connected kinase hubs together with bridge proteins linking the kinase and metabolic enzyme layers, providing a systems-level view of kinase association patterns across glucose metabolism. Together, these findings indicate that kinase associations are not uniformly distributed across glucose metabolism but preferentially concentrate at metabolic control points that govern pathway routing and glucose allocation. This study provides a systems-level framework for prioritizing candidate kinase–enzyme associations and a resource for investigating signaling–metabolism interactions in metabolic disease and cancer.

## 1. Introduction

Glucose metabolism lies at the core of cellular physiology, serving as a central hub that integrates energy production, biosynthesis, and redox balance across diverse biological systems. Within cells, glucose-derived carbon is processed through an interconnected network of pathways, including glycolysis and gluconeogenesis, glycogen metabolism, the pentose phosphate pathway, and auxiliary routes such as galactose, fructose, and mannose metabolism, that collectively determine whether glucose is utilized for ATP generation, stored as glycogen, or redirected toward biosynthetic and signaling functions. These pathways are linked through shared metabolites, with intermediates such as glucose-1-phosphate, glucose-6-phosphate, fructose-6-phosphate, and pyruvate acting as metabolic branch points that partition carbon among multiple cellular fates [1,2,3,4]. The behavior of this network is determined by metabolic enzymes that catalyze each reaction step, with branch-point enzymes directing carbon allocation between competing pathways and irreversible enzymes governing metabolic flux through key reactions. In this context, glucose metabolism is controlled largely by a set of “gatekeeper” enzymes, which are tuned at multiple levels, including allosteric regulation, phosphorylation, subcellular localization, and gene expression, to direct glucose toward ATP production, storage, or biosynthesis while maintaining metabolic balance [5,6]. Together, these enzymes establish the metabolic control architecture that enables cells to dynamically balance energy production, storage, and biosynthesis. However, this raises a fundamental question: how is the activity of these enzymes themselves regulated to enable dynamic and context-dependent control of metabolism?

In multicellular organisms, enzyme activity is tightly coordinated by signaling networks that integrate extracellular and intracellular cues to maintain metabolic homeostasis. Cells sense hormones, growth factors, and cytokines through receptor-mediated pathways that activate intracellular cascades, in which protein kinases play a central role. Protein kinases phosphorylate specific residues on target proteins, altering enzyme activity, stability, localization, or protein interactions, providing a direct mechanism to regulate metabolic reactions [7,8,9,10]. For example, nutrient-responsive kinases such as ULK1/2 coordinately phosphorylate multiple glycolytic and gluconeogenic enzymes to redirect glucose metabolism during nutrient stress, whereas Aurora A-mediated phosphorylation of PKM2 promotes the accumulation of glycolytic intermediates that support anabolic metabolism in cancer cells [11,12]. Notably, metabolic enzymes are subject to diverse post-translational modifications, but phosphorylation represents a major regulatory layer capable of dynamically altering enzymatic function [13]. Through such modifications, kinase activity has the potential to reshape metabolic flux by acting on enzymes that control key steps in metabolic pathways [14]. The composition and activity of kinase signaling networks are also influenced by transcriptional and epigenetic regulation of kinase-encoding genes, contributing to context-dependent phosphorylation patterns observed in physiology and disease [15]. These upstream regulatory mechanisms further contribute to the dynamic nature of kinase signaling across different biological contexts. Nevertheless, numerous kinase–substrate relationships involving metabolic enzymes have been reported under diverse biological conditions, yet how these associations are organized across the glucose metabolic network remains largely unknown. Elucidating this is therefore essential for understanding how signaling pathways influence metabolite dynamics in complex biological systems. A major challenge is that, unlike experimentally established enzyme–metabolite relationships [16], many reported kinase–enzyme associations originate from heterogeneous kinase–substrate databases, large-scale phosphoproteomic inference studies, and sequence-based prediction methods, resulting in extensive but variable evidence supporting individual associations [17,18,19,20]. While these resources have substantially expanded the known kinase–substrate landscape, they are rarely interpreted within the structural organization of metabolic networks. Consequently, the large number of reported kinase associations may obscure those that are consistently supported across multiple lines of evidence, making it difficult to distinguish biologically plausible kinase–enzyme associations from prediction-driven assignments. These limitations have motivated the development of computational approaches for kinase activity inference, kinase–substrate prediction, and phosphoproteomic network analysis.

Accordingly, recent computational methods have substantially advanced kinase biology through kinase activity inference, kinase–substrate prediction, phosphoproteomic enrichment analysis, and machine-learning-based prediction frameworks [21,22,23,24]. These approaches have improved the interpretation of phosphoproteomic datasets and expanded kinase–substrate resources. However, they primarily focus on predicting kinase–substrate relationships or inferring kinase activity from phosphorylation data. By contrast, comparatively little attention has been given to understanding how kinase associations are organized within metabolic pathway architecture. Consequently, the systems-level organization of kinase associations across glucose metabolism remains largely unexplored. An important unresolved question is whether prioritized kinase associations exhibit non-random organization across the glucose metabolic network. Specifically, it remains unclear whether kinase associations preferentially target metabolic branch points, flux-controlling reactions, or enzymes integrating multiple control functions, and whether these associations are organized through higher-order network features such as highly connected kinase hubs and bridge proteins linking signaling and metabolic networks. Addressing these questions requires integrating signaling information with metabolic network architecture to move beyond individual kinase–substrate relationships toward a network-level understanding of kinase association patterns across glucose metabolism. In this study, we developed a multi-layer framework to systematically prioritize kinase–metabolic enzyme associations across glucose metabolism by integrating curated kinase–substrate annotations, phosphoproteomic co-regulation, and protein–protein interaction networks. We hypothesized that integrating these complementary evidence layers would enable the identification of biologically relevant kinase–enzyme relationships beyond those supported by individual data sources alone. Using this framework, we ranked kinase associations according to their combined evidence strength and investigated how prioritized associations were organized across glucose metabolic pathways, metabolic control points, and the overall network architecture. We further identified highly connected kinase hubs and bridge proteins linking kinase signaling with metabolic enzymes within the glucose metabolic network. Collectively, this study provides a systems-level resource for prioritizing candidate kinase–enzyme associations and investigating how kinase-mediated regulation is organized across glucose metabolism. The overall biological rationale and analytical workflow of the present study are summarized in Figure 1.

## 2. Results

### 2.1. Mapping Kinase–Metabolic Enzyme Associations Across Glucose Metabolism

We reconstructed a glucose metabolic network comprising 144 metabolic enzymes across core glucose utilization pathways together with associated carbohydrate conversion and dietary carbohydrate entry pathways, including glycolysis/gluconeogenesis, the pentose phosphate pathway, glycogen metabolism, fructose and mannose metabolism, galactose metabolism, and starch and sucrose metabolism (Figure 2A, Supplementary Table S1). Pathway overlap analysis showed that 27 enzymes participated in two or more pathways, reflecting the interconnected nature of glucose metabolism, in which individual enzymes contribute to multiple metabolic processes simultaneously (Figure 2B). Compilation of phosphorylation annotations from dbPTM [25], qPTM [26], and PhosphoSitePlus [27] identified 4093 phosphorylation sites across the 144 enzymes, with phosphorylation site counts ranging from 1 to 86 per enzyme (mean = 28.4 sites/enzyme). Serine phosphorylation represented the dominant modification type (68.4%), followed by threonine (26.1%) and tyrosine phosphorylation (5.5%). Several enzymes, including GYS1 (80 sites), HK1 (73 sites), GBE1 and PKM (70 sites), harbored particularly large numbers of annotated phosphosites, indicating extensive post-translational regulatory potential within central glucose metabolism.

Integration of data from the current kinase–substrate databases identified kinase associations for 970 phosphosites across 138 metabolic enzymes, generating 25,640 kinase–substrate–site relationships involving 444 unique kinases. These assignments originated from multiple evidence sources, including curated low-throughput experimental databases, high-throughput kinase profiling datasets, large-scale phosphoproteomic inference resources, and sequence-based prediction methods. Curated experimental evidence from PhosphoSitePlus [27], Phospho.ELM [28], and RegPhos [29] accounted for 172 relationships (0.77%), while high-throughput kinase profiling data from iKiP-DB [30] contributed 575 relationships (2.58%). Large-scale phosphomotif mutant-based inference resources, including Ochoa et al. (2020) [31] and Johnson et al. (2023) [20], accounted for 15,503 relationships (69.54%), whereas sequence-based prediction approaches such as NetworKIN [32] and AKID [33] contributed 6895 relationships (30.93%). The majority of assignments were derived from large-scale phosphoproteomic inference and prediction-based resources, rather than direct low-throughput experimental validation, indicating that many kinase–substrate associations remain computationally inferred rather than directly validated. Hence, to prioritize kinase associations supported by biological confidence, kinase–substrate pairs were further evaluated using phosphoproteomic co-regulation analysis across 154 published phosphoproteomic datasets. Co-regulation was defined by coordinated differential expression of phosphosites in metabolic enzyme(s) and kinase(s) across experimental conditions relative to their respective control conditions. Following co-regulation filtering, 6161 kinase–substrate relationships remained supported, corresponding to approximately 24% of the initial assignments. This reduction indicates that only a subset of annotated kinase–substrate relationships showed potential reproducible phosphoproteomic associations across the currently explored phosphoproteomics datasets and experimental conditions.

To extend the confidence of the analysis beyond potential direct kinase–substrate relationships, protein–protein interaction data were integrated from HPRD [34], BioGRID [35], ConsensusPathDB [36], CORUM [37], BIND [38], and RegPhos [29]. These shared interacting proteins were incorporated to capture potential scaffold-mediated proximity and complex-associated regulatory mechanisms through which kinases may indirectly influence metabolic enzyme function.

Integration of protein–protein interaction datasets identified 9192 proteins shared between kinases and glucose metabolic enzymes, providing the interaction layer used for subsequent tripartite network analyses. Subsequent integration of phosphoproteomic co-regulation with this interaction layer identified 54,844 substrate–interactor co-regulation pairs and 489,791 kinase–interactor co-regulation pairs. Together, the direct kinase–substrate and interactor-supported co-regulation layers incorporated evidence from 191 independent phosphoproteomic studies. Integration of primary kinase–substrate assignments, phosphoproteomic co-regulation, and interactor-supported relationships resulted in a multi-layer glucose kinase association network incorporating both direct kinase–substrate associations and indirect interaction-supported relationships, providing the foundation for subsequent kinase prioritization and systems-level analysis of glucose metabolic regulation.

### 2.2. Prioritization of Phosphoproteomic-Supported Kinase Associations Across Glucose Metabolism

To reduce the large number of kinase associations obtained from integrated kinase–substrate resources into a more biologically interpretable set of candidate kinase associations, kinase associations were prioritized using a composite scoring framework integrating direct database evidence, phosphoproteomic co-regulation, and interaction-supported co-regulation evidence (Supplementary Table S2). Kinase associations were ranked according to the combined strength of evidence across these complementary data layers, and high-confidence associations were retained using score- and phosphoproteomic-support-based prioritization criteria. Application of this prioritization framework substantially reduced the initial kinase association space while retaining broad pathway coverage across glucose metabolism. The prioritized dataset contained 24,060 kinase–substrate associations involving 278 kinases across 121 metabolic enzymes distributed among glycolysis/gluconeogenesis, the pentose phosphate pathway, glycogen metabolism, fructose and mannose metabolism, galactose metabolism, and starch and sucrose metabolism. Kinases retained after prioritization showed higher contributions from phosphoproteomic co-regulation evidence compared to the initial unfiltered assignment space, indicating the preferential retention of kinase associations reproducibly supported across independent phosphoproteomic datasets.

Pathway-level analysis of prioritized associations revealed substantial differences in kinase association density across glucose metabolic branches after normalization to pathway size (Figure 3A). Glycogen metabolism showed the highest kinase association density, with 227 kinase–substrate pairs per enzyme, compared with 139 pairs per enzyme in glycolysis/gluconeogenesis and 41 pairs per enzyme in starch and sucrose metabolism (Figure 3B,C). Although glycolysis/gluconeogenesis contained the largest number of enzymes and total kinase associations, smaller pathways such as glycogen metabolism maintained comparatively high kinase association densities through concentrated associations distributed across fewer enzymes. In contrast, starch and sucrose metabolism showed the lowest kinase association density across all analyzed pathways. Despite differences in pathway-level kinase association density, enzyme-level association scores were broadly similar across pathways. Pairwise comparisons showed no significant differences after multiple-testing correction, indicating that the pathways differed mainly in the number of prioritized kinase associations per enzyme rather than in large differences in phosphoproteomic-supported association strength.

To evaluate pathway-associated kinase composition independent of pathway size, pathway preference analysis was performed using normalized observed/expected association ratios at both the kinase-group and kinase-family levels (Figure 3D and Figure S1). Most kinase groups and families showed pathway preference values close to the global network background, indicating broad distribution across multiple glucose metabolic branches rather than strong pathway restriction. Glycogen metabolism showed relatively higher representation of AGC-, CK1-, and Atypical-group kinases, including the GSK, PDHK, RSK, MAPKAPK, and CK1 families. In contrast, galactose metabolism and starch and sucrose metabolism showed comparatively higher representation of several tyrosine kinase-associated families, including Met-, Src-, Abl-, Eph-, FGFR-, and EGFR-related kinases, together with CMGC-associated families such as CLK, DYRK, and SRPK. Fructose and mannose metabolism exhibited a modest preference for kinase families associated with the AGC and CMGC groups, including Akt-, PKA-, SGK-, DYRK-, and SRPK-related kinases. Glycolysis/gluconeogenesis and the pentose phosphate pathway showed comparatively broad kinase-family distributions, with recurrent associations distributed across MAP kinase-associated families, STK39, MAP4K4, IRAK1, and NEK-family kinases. In addition, several kinases, including PRKAA1, MTOR, CAMK2D, PKN2, and WNK1, showed broad association patterns across nearly all analyzed pathways together with relatively high phosphoproteomic-supported association fractions (Figure S2). Collectively, these findings indicate that although prioritized kinase associations are widely distributed throughout glucose metabolism, subsets of kinase groups and families exhibit reproducible pathway-associated composition patterns within specific metabolic branches.

Prioritized kinase associations revealed substantial variation at the individual enzyme level. To identify enzymes with high densities of phosphoproteomic-supported kinase associations, enzymes were ranked using an association density score integrating the number of associated kinases, mean phosphoproteomic co-regulation support, and phosphosite-normalized kinase association density. PDHA1 emerged as the highest-ranking enzyme, exhibiting 158 unique kinase associations across 12 retained phosphosites together with the highest mean phosphoproteomic co-regulation support among all analyzed enzymes. Several additional high-ranking enzymes were associated with key branch and flux-control steps, including PFKFB3, PFKFB2, PFKP, and GYS1. These enzymes showed elevated kinase association density scores despite relatively modest phosphosite numbers, indicating concentrated phosphoproteomic-supported kinase association patterns (Figure S3). In contrast, several broadly studied glycolytic enzymes, including HK1, showed comparatively low association density scores due to the limited phosphoproteomic-supported associations retained after prioritization.

Together, these analyses define a refined phosphoproteomic-supported kinase association landscape across glucose metabolism, characterized by uneven pathway-level association density, pathway-associated kinase composition patterns, and the preferential accumulation of high-ranking kinase associations on enzymes involved in glucose allocation and metabolic decision points.

### 2.3. Kinase Association Patterns at Glucose Metabolic Decision Points

Having established phosphoproteomic-supported kinase associations across glucose metabolism, we examined whether kinase association density varies with metabolic control architecture. Metabolic flux is governed by two architectural features: (i) branch points, where a metabolite feeds multiple competing pathways, and (ii) flux-controlling steps, where thermodynamically irreversible reactions commit substrates to specific fates. We classified glucose metabolic enzymes into five functional categories: dual-control (enzymes that are both branch points AND flux-controlling (*n* = 9), branch-only (*n* = 22), flux-only (*n* = 7), opposing (gluconeogenic; *n* = 7), and non-control (*n* = 89).

Using high-confidence kinase–substrate associations (Rank ≤ 10, phosphoproteomic fraction ≥ 15%; 6247 pairs across 134 enzymes), we compared kinase association density across functional categories. Dual-control enzymes exhibited the highest total number of kinase associations among all categories (mean = 203.8 vs. 81.3 for non-control; fold difference = 2.5; Mann–Whitney *p* = 0.0004), followed by opposing (mean = 170.57, *p* = 0.0044), flux-only (mean = 135.8, *p* = 0.0124) and branch-only (mean = 120.8, *p* = 0.0109) enzymes, whereas non-control enzymes showed the lowest values, establishing a consistent ordering of dual-control > opposing > branch-only ≈ flux-only > non-control (Figure 4A). Because total kinase association counts may be influenced by phosphosite abundance, kinase associations were additionally normalized by phosphosite number. Following normalization, the separation between categories was reduced and no longer globally significant (Kruskal–Wallis *p* = 0.11), although dual-control and branch-associated enzymes continued to show comparatively elevated kinase associations per phosphosite relative to non-control enzymes (Figure 4B). Among individual enzymes, PKM and GYS1 exhibited the highest absolute numbers of high-confidence kinase associations and phosphosites, while PDHA1, GAPDH, ALDOA, PGK1, and LDHA showed comparatively high phosphoproteomic-supported kinase association densities.

#### 2.3.1. Kinase Association Patterns Across Branch Metabolites

Kinase associations varied across the five glucose metabolic branch points. We analyzed mean kinase input, phosphorylation support, and high-priority kinase pairs for enzymes at each branch point. In terms of total high-priority pairs, fructose-6-phosphate ranked highest (1724 high-priority substrate–site–kinase pairs across 9 enzymes and 166 kinases) with strongest mean phosphorylation support (19.3% mean phospho fraction), followed by glucose-6-phosphate, while pyruvate showed the lowest (Figure 4C).

#### 2.3.2. Shared and Distinct Kinase Association Patterns Across Futile Cycling Enzyme Pairs

We examined kinase association patterns across four opposing enzyme pairs that interconvert the same metabolites in the forward (glycolytic/anabolic) and reverse (gluconeogenic/catabolic) directions. Kinase association overlap varied substantially across pairs (Figure 4D):

HK vs. G6PC (glucose phosphorylation ↔ dephosphorylation): High-confidence kinase interactions were detected exclusively for hexokinases, with no corresponding interactions for G6PC enzymes under the high-confidence criteria (13.0 vs. 0.0 mean high-confidence kinases per enzyme; Jaccard similarity = 0, indicating complete kinase separation).

PFK vs. FBP (F6P ↔ F1,6BP commitment): PFK enzymes showed substantially higher high-confidence kinase input compared to FBP enzymes (45.7 vs. 17.0 mean high-confidence kinases per enzyme), indicating a mixed pattern of kinase sharing (Jaccard = 0.20), with 41 kinases shared and 82 PFK-specific.

PK vs. PEPCK/PC (PEP ↔ pyruvate): Both forward and reverse enzymes showed enrichment of high-confidence interactions, with stronger targeting of PK enzymes (70.5 vs. 19.0 mean high-confidence kinases per enzyme). The PK–PEPCK/PC pair showed the most pronounced kinase asymmetry, with 103 kinases being forward-specific versus only 20 being reverse-specific, despite 25 kinases being shared between directions.

GYS vs. PYG (glycogen synthesis/breakdown): Under total kinase counts, glycogen phosphorylase (PYG) showed more kinase associations than glycogen synthase (258.7 vs. 201.5 mean kinases per enzyme). Under high-confidence filtering, the pattern reversed (68.5 GYS vs. 39.7 PYG), and GYS and PYG shared the majority of their high-confidence kinases (Jaccard = 0.30; 63 shared kinases), indicating extensive overlap in kinase association profiles between the synthesis and breakdown directions.

Across all pairs, enzymes catalyzing the forward direction showed higher high-confidence kinase input than their reverse counterparts (Figure S4). Kinase sharing patterns ranged from complete separation (HK/G6PC) through partial overlap (PFK/FBP and PK/PEPCK) to extensive co-regulation (GYS/PYG), indicating variable degrees of kinase association overlap across opposing metabolic reactions.

#### 2.3.3. Kinase Association Density Scoring Identifies Enzymes with Concentrated Kinase Associations

Under the composite association density score metric, PDHA1 showed the highest overall association density score (2.83), followed by GYS1 (2.41), PFKP (2.19), and PFKL (1.65). Several of the highest-ranking enzymes occupied major metabolic branch or flux-decision positions, including enzymes associated with glycogen metabolism, glycolytic commitment, the pentose phosphate pathway entry, and mitochondrial carbon entry.

Among branch-associated enzymes, GPI (1.64), PGM2L1 (1.62), TALDO1 (1.46), GAPDH (1.30), and ALDOA (1.08) showed comparatively elevated association density scores. In contrast, several enzymes with large absolute numbers of kinase associations, including HK1, HK2, FBP1, and GYS2, showed lower association density scores after phosphoproteomic-supported prioritization, reflecting the reduced contribution from co-regulation-supported associations within the filtered dataset. Thus, association density scoring distinguished enzymes with concentrated phosphoproteomic-supported kinase convergence from enzymes with broader but less strongly supported kinase association profiles (Supplementary Table S3).

### 2.4. Network-Level Organization of Kinase–Enzyme Associations Across Glucose Metabolism

To characterize the global organization of kinase–enzyme associations across glucose metabolism, we constructed a bipartite network comprising 297 kinases and 117 metabolic enzymes connected by 2785 high-confidence associations (Rank ≤ 10 per enzyme; phosphoproteomic support fraction ≥ 15%; network density = 0.033). The resulting network exhibited a heterogeneous degree distribution, with a small subset of kinases associated with disproportionately large numbers of metabolic enzymes (maximum degree = 63; mean degree = 9.4).

We defined kinase “hubs” operationally as kinases with a degree ≥ 2× the network mean (threshold = 18.75 targets), identifying 41 hubs (13.8% of network kinases) that collectively connect to 92.3% of the 117 glucose metabolic enzymes in the network (Figure 5). The five highest-degree kinases were LYN (63 enzyme associations, Src family), ABL1 (50, Abl), LCK (49, Src), SRC (48, Src), and YES1 (47, Src). These kinases associated with a broad cross-section of metabolic enzyme types, including branch-point enzymes (18–20 each), flux-control enzymes (8–11 each), and dual-control enzymes (6–7 each). Notably, PRKAA1 (AMPKα1), a well-established energy-sensing kinase, ranked among the top 10 hubs (degree = 40), and GSK3B, a known regulator of glycogen synthase, was identified as a hub (degree = 22), consistent with the known roles of these kinases in glucose metabolism. To assess whether particular kinase families are disproportionately represented among hubs, we performed hypergeometric enrichment analysis comparing family membership among hubs with that in the full network of 297 kinases (22 families tested). After Benjamini–Hochberg FDR correction, the Src family showed significant enrichment (6/11 Src kinases were classified as hubs; 4.0-fold enrichment; FDR-adjusted *p* = 0.029). Additional families, including Abl (7.2-fold), MAST (5.4-fold), CK1 (4.3-fold), and RSK (3.6-fold), showed nominal enrichment (uncorrected *p* < 0.05) but did not remain significant after multiple-testing correction (Supplementary Table S4). Nevertheless, these families exhibited higher hub representation than expected from their overall frequency within the network.

Enzyme in-degree analysis (the number of distinct kinases associating with each enzyme) identified a subset of highly connected metabolic enzymes. The ten most-connected enzymes were PKM (126 kinase associations, 23% from hubs), GYS1 (117, 27%), ENO1 (104, 26%), GAPDH (102, 27%), ALDOA (96, 33%), PGAM1 (76, 33%), PGK1 (76, 37%), TKT (73, 30%), LDHA (71, 35%), and PFKP (66, 33%). Among these, control-point enzymes (branch, flux-control, or dual-control) accounted for seven of the top ten, consistent with, though not sufficient to establish, a model in which kinase association density is elevated at metabolic nodes that govern pathway routing and flux distribution. ENO1 and PGAM1, although classified as non-control enzymes in the current framework, were also among the most highly connected enzymes.

Beyond direct kinase–enzyme associations, integration of protein–protein interaction data identified 567 bridge proteins connecting kinases and metabolic enzymes within the tripartite network. Bridge proteins were ranked using a composite metric incorporating bridge score and betweenness centrality. Several of the highest-scoring proteins corresponded to broadly connected cellular hubs, including members of the HSPA family, tubulins, and GRB2. In addition, a subset of highly ranked bridge proteins with established links to cellular signaling and metabolism was identified, including TFAM, CYLD, EIF4B, TRIM28, and CAD. These proteins connected multiple kinases to branch-point, flux-control, and dual-control enzymes and occupied central positions within the tripartite network (Supplementary Table S5 and Figure S5).

Collectively, these analyses reveal a network organization in which a relatively small subset of high-connectivity kinases accounts for a large proportion of kinase–enzyme associations across glucose metabolism. In addition, tripartite network analysis identified a subset of highly connected bridge proteins linking the kinase and metabolic enzyme layers, extending the network beyond direct kinase–enzyme associations.

## 3. Discussion

Cells continuously decide whether glucose is directed toward energy production, storage, or biosynthesis, and these metabolic outcomes depend on the coordinated regulation of the enzymes that govern glucose metabolism [39,40]. Glucose metabolism was selected as the focus of this study because it occupies a central position in cellular metabolism, linking glycolysis, glycogen metabolism, the pentose phosphate pathway, gluconeogenesis, and the tricarboxylic acid cycle, while many of its key enzymes are regulated by reversible phosphorylation. In this study, we investigated how kinase–enzyme associations are organized across the human glucose metabolic network and whether these associations align with established principles of metabolic control. To address this, we developed a multi-layer framework integrating kinase–substrate annotations, phosphoproteomic co-regulation, and protein–protein interaction networks to prioritize candidate kinase–enzyme associations supported by complementary evidence. The resulting phosphoproteomic-supported network demonstrated that prioritized kinase associations are not uniformly distributed across glucose metabolism but are preferentially concentrated at metabolic control points, particularly enzymes that integrate branch-point and flux-control functions. Furthermore, network analysis identified a relatively small subset of highly connected kinase hubs together with bridge proteins linking the kinase and metabolic enzyme layers. Collectively, these findings provide a systems-level view of how kinase associations are organized within glucose metabolism and establish a framework for prioritizing candidate kinase–enzyme associations for future experimental investigation. Although the present study focuses on glucose metabolism, the proposed framework is readily applicable to other metabolic pathways.

Unlike existing computational approaches that primarily infer kinase activity or predict kinase–substrate relationships, our framework integrates complementary evidence to prioritize supported kinase–enzyme associations within their metabolic network context. Current kinase–substrate resources combine curated databases, high-throughput phosphoproteomic studies, and computational prediction, resulting in extensive but heterogeneous collections of reported kinase–substrate relationships. Consequently, many annotated kinase–enzyme associations remain context-dependent and are not consistently supported across independent experimental datasets [24,41,42]. Phosphoproteomic data provide an important complementary evidence layer by capturing coordinated phosphorylation dynamics observed across diverse biological conditions [43,44]. Building on this, our framework combines kinase–substrate annotations with phosphoproteomic co-regulation and protein–protein interaction evidence to rank kinase–enzyme associations according to the combined supporting evidence rather than treating all reported interactions equally. This approach substantially reduced the initial association space while retaining broad coverage across the glucose metabolic network, thereby providing a more biologically interpretable set of candidate kinase–enzyme associations for downstream analyses.

Most previous studies investigating kinase-mediated phosphorylation of glucose metabolism have focused on individual enzymes or specific metabolic pathways. For example, PLK1-mediated phosphorylation of PDHA1 suppresses pyruvate entry into the tricarboxylic acid cycle and promotes glycolytic reprogramming in cancer, whereas Src-mediated phosphorylation of HK1 and HK2 enhances glycolytic activity by increasing hexokinase function [45,46,47]. Beyond glycolytic entry, Src signaling has also been implicated in regulating additional metabolic enzymes, including PFKFB3, LDHA, and G6PD, together with signaling pathways involving HIF-1α, c-Myc, and PI3K/Akt that collectively influence glucose utilization and anabolic metabolism [48]. These studies demonstrate that kinase-mediated phosphorylation can influence multiple components of glucose metabolism; however, they have primarily focused on individual enzymes or discrete metabolic modules rather than the organization of kinase associations across the metabolic network. In contrast, our study adopts a metabolism-centric perspective by systematically prioritizing kinase–enzyme associations across the entire glucose metabolic network, enabling kinase association patterns to be interpreted within the context of metabolic topology rather than isolated enzymatic reactions.

Among individual pathways, glycogen metabolism emerged as the most prominent example of this organizational pattern. Despite containing relatively few enzymes, it exhibited the highest pathway-level kinase association density, and glycogen synthase (GYS1) ranked among the highest-scoring enzymes based on phosphoproteomic-supported kinase association density. These observations are consistent with the established role of glycogen metabolism as a major metabolic decision point that integrates hormonal and energetic signals controlling glucose storage and utilization. Indeed, kinases such as GSK3, AMPK, and PKA are well-established regulators of GYS1 phosphorylation and glycogen synthesis under conditions of hormonal stimulation and energy stress [49,50,51,52]. In addition, signaling pathways involving focal adhesion kinase (FAK) have been reported to influence glycogen synthesis through modulation of Akt and GSK3β signaling [53]. Interestingly, although kinase-mediated phosphorylation has been studied extensively in the context of glycolysis and cancer-associated metabolic reprogramming [54,55,56], our network-level analysis identified glycogen metabolism as the pathway exhibiting the greatest concentration of prioritized kinase associations. Together, these observations place our findings within the context of existing experimental evidence while highlighting glycogen metabolism as a prominent signaling-associated component of the glucose metabolic network.

A central finding of this study is that prioritized kinase associations were preferentially concentrated at metabolic control points rather than being distributed uniformly across the glucose metabolic network. Enzymes integrating both branch-point and flux-control functions exhibited the highest kinase association density, followed by branch-point and flux-control enzymes, whereas other metabolic enzymes generally showed lower levels of kinase association. This organization is consistent with classical principles of metabolic control, in which relatively few enzymatic steps exert a disproportionate influence over pathway routing and metabolic flux [2,57]. Although our analyses do not establish direct regulatory mechanisms, they suggest that kinase associations preferentially accumulate at enzymes occupying key positions within the metabolic network. Enzyme-level analyses also suggest distinct patterns of kinase association among metabolic control-point enzymes. Dual-control enzymes tended to accumulate kinase associations across multiple phosphorylation sites, whereas several branch-point enzymes exhibited relatively high kinase association density despite fewer retained phosphosites. Although these patterns do not establish distinct mechanisms of kinase action, they suggest that different classes of metabolic control-point enzymes may integrate kinase associations through different organizational strategies, warranting future phosphosite-resolved functional studies.

Beyond the pathway level, this organization becomes particularly important in futile cycles, where opposing reactions must be tightly coordinated to prevent simultaneous forward and reverse flux that would otherwise waste ATP [58,59]. We observed a strong asymmetry in kinase targeting, with forward reactions receiving substantially greater numbers of prioritized kinase associations, consistent with classical substrate cycle theory predicting directional bias for metabolic efficiency [60]. However, different futile-cycle pairs employed distinct coordination strategies depending on the metabolic context. Complete separation of kinase associations, as observed for hexokinase and glucose-6-phosphatase, likely reflects compartmentalization and tissue specificity that inherently minimize futile cycling [61]. In contrast, glycogen synthesis and breakdown exhibited high kinase sharing, enabling coordinated switching between storage and mobilization. Other futile cycles exhibited intermediate coordination patterns balancing shared and pathway-specific regulation. Notably, the pyruvate kinase–PEPCK pair emerged as a key bidirectional control node linking glycolysis, gluconeogenesis, and TCA cycle entry [62]. Together, these observations suggest that patterns of prioritized kinase associations differ among futile cycles according to their metabolic context, with distinct balances of shared and reaction-specific kinase associations across individual reaction pairs.

The organization of kinase associations at metabolic control points raises an important question: how are these local association patterns related across the broader glucose metabolic network? Our network analysis identified a relatively small subset of highly connected hub kinases that were associated with a large proportion of metabolic enzymes, indicating that kinase–enzyme associations are organized around a limited number of broadly connected signaling proteins rather than being distributed uniformly across the network. In particular, 41 hub kinases were associated with more than 90% of the glucose metabolic enzymes included in the network. Src- and Abl-family kinases were significantly overrepresented among these hubs, consistent with their well-established roles in growth factor signaling and metabolic adaptation [63,64,65]. AMPK (PRKAA1), a central cellular energy sensor, also emerged as one of the highly connected hub kinases, consistent with its established role in coordinating cellular responses to energetic stress [66,67,68]. Together, these observations suggest that a relatively small number of highly connected kinases contribute substantially to the organization of kinase associations across the glucose metabolic network.

Our analysis further revealed that kinase–enzyme regulation extends beyond direct phosphorylation through an additional layer mediated by bridge proteins. These proteins may facilitate indirect regulation by organizing kinase–enzyme proximity or by transmitting phosphorylation-dependent effects through enzyme-associated protein complexes. Although many highly ranked bridge proteins, including members of the HSP family and cytoskeletal proteins, represent broadly connected cellular interactors, several candidates such as TRIM28, TFAM, CYLD, and CAD have established roles in cellular signaling or metabolism and occupied central positions within the tripartite network [69,70,71,72,73,74]. For example, TRIM28 has been implicated in regulating LDHA expression and glycolytic activity following RSK-dependent phosphorylation [75], supporting its potential relevance as a bridge between signaling and metabolic pathways. Together with the identified hub kinases, these findings indicate that kinase–enzyme associations are organized at multiple network levels, encompassing direct kinase–enzyme relationships, highly connected hub kinases, and shared interacting proteins. Rather than existing as isolated phosphorylation events, kinase–substrate associations form interconnected network structures that can be interpreted within the context of metabolic topology. This systems-level organization complements traditional pathway-centric studies and provides a framework for prioritizing candidate signaling modules linking kinase signaling with glucose metabolism for future experimental investigation.

The prioritization framework presented here provides a systematic approach for identifying biologically supported kinase–enzyme associations that warrant further experimental investigation in metabolic disease and cancer. Rather than focusing on individual metabolic enzymes, the framework enables the prioritization of highly connected hub kinases, metabolic control-point enzymes, and bridge proteins that may represent key regulatory nodes linking signaling and metabolism. By integrating complementary evidence from kinase–substrate annotations, phosphoproteomic co-regulation, and protein interaction networks, the proposed approach generates a biologically supported resource for selecting candidate kinase–enzyme associations for targeted experimental validation and mechanistic studies.

Several limitations should be considered when interpreting these findings. The study relies on currently available phosphoproteomic and kinase–substrate datasets, which remain biased toward well-characterized kinases and frequently studied experimental systems. Consequently, less-studied kinases and context-specific kinase–enzyme associations are likely to be underrepresented. Second, although integration of kinase annotations, phosphoproteomic co-regulation, and protein interaction networks increases confidence in prioritized kinase–enzyme associations, the framework does not establish direct kinase–substrate interactions or causal mechanisms. Accordingly, the prioritized associations should be regarded as biologically supported candidates requiring validation through targeted experimental approaches, including phosphosite-specific functional assays, kinase perturbation studies, phospho-Western analyses, or metabolomic measurements linking phosphorylation changes to metabolic outcomes. Furthermore, the network presented here represents an aggregate view across multiple datasets and conditions, whereas kinase signaling is inherently dynamic and context-dependent. An important limitation underlying current network-based studies is the limited availability of synchronized multi-omics datasets in which metabolomics, proteomics, phosphoproteomics, and transcriptomics are measured simultaneously within the same biological system. Future studies integrating tissue-specific, temporal, and condition-resolved multi-omics datasets together with targeted experimental validation of prioritized kinase–enzyme associations, hub kinases, and bridge proteins will be essential for defining causal signaling–metabolism relationships. Future context-resolved analyses incorporating environmental and epigenetic influences, as well as application of the framework to disease-specific phosphoproteomic and multi-omics datasets, may further reveal how kinase–metabolic enzyme associations are reorganized across physiological and pathological conditions.

## 4. Materials and Methods

### 4.1. Compilation of Human Glucose Metabolic Enzymes and Phosphorylation Sites

Human glucose metabolic enzymes were compiled from KEGG pathway annotations (Release 115.0) [16], including glycolysis/gluconeogenesis (hsa00010), the pentose phosphate pathway (hsa00030), fructose and mannose metabolism (hsa00051), galactose metabolism (hsa00052), and starch and sucrose metabolism (hsa00500). Enzyme names were harmonized with HGNC gene symbols (last updated: 5 May 2026) and mapped to canonical UniProt protein accessions (Release 2025_04) [76]. Multi-pathway enzymes were assigned to all pathways in which they participate.

Experimentally validated phosphorylation sites corresponding to metabolic enzymes were retrieved from PhosphoSitePlus (v6.7.5) [27], dbPTM (Release 2022) [25], and qPTM [26] (Release 2023). All phosphosites were remapped to canonical UniProt reference sequences to ensure uniform residue mapping. Redundant entries across databases were merged based on site, residue and modification types. Subsequently, serine, threonine, and tyrosine phosphorylation sites were retained for downstream analyses.

### 4.2. Assembly of Kinase–Substrate–Site Associations

Kinase–substrate relationships for metabolic enzyme phosphosites were assembled from experimentally validated databases, high-throughput kinase screening resources, and computational kinase prediction tools. Experimentally validated kinase–substrate associations were collected from PhosphoSitePlus [27], Phospho.ELM [28], and RegPhos [29]. High-throughput kinase profiling data were obtained from iKiP-DB [30], and kinase–substrate prediction datasets derived from large-scale phosphopeptide screening studies reported by Johnson et al. (2023) [20] and Ochoa et al. (2020) [31]. Predicted kinase–substrate relationships were further obtained using the NetworKIN [32] and AKID [33] tools. All enzymes, kinases and their phosphosites were harmonized to HGNC gene symbols and canonical UniProt accessions. Duplicate kinase–substrate–site associations were removed while preserving source-specific evidence-based annotations. Each interaction was classified according to the following evidence types:

(i) Experimentally validated;
(ii) High-throughput;
(iii) Phosphosite inference-based;
(iv) Computationally predicted.

The resulting kinase–substrate–site triplet dataset served as the primary input for kinase prioritization analyses.

### 4.3. Curation of Quantitative Human Phosphoproteomics Datasets

Human phosphoproteomics datasets were curated through systematic PubMed searches using the terms “phosphoproteomics” OR “phosphoproteome”, excluding review articles and studies on plant materials. Studies oriented toward quantitative differential phosphorylation profiling between defined biological conditions and controls were considered. A total of 154 quantitative human phosphoproteomics studies (or datasets) were retained after filtering. To ensure high-confidence site identification, Class-I phosphosites (localization probability ≥ 75% or A-score ≥ 13) were retained. Phosphosites were classified as upregulated or downregulated based on study-specific quantitative thresholds (fold change ≥ 1.3 or ≤ 0.76; *p* < 0.05). All phosphosites were mapped to canonical UniProt sequences and standardized to HGNC symbols. Experimental conditions were harmonized across datasets to facilitate cross-study comparisons. Considering that the datasets originated from heterogeneous experimental platforms and normalization strategies, analyses were performed using the reported differential phosphorylation values without raw-data reprocessing.

### 4.4. Phosphosite Co-Regulation Analysis

Phosphosite-level co-regulation analysis was performed using curated quantitative phosphoproteomics datasets. For each metabolic enzyme phosphosite, datasets were grouped according to whether the site was upregulated or downregulated under a given condition. Phosphosites in kinases or interacting proteins were classified as:

- Concordantly regulated (up–up or down–down);
- Discordantly regulated (up–down or down–up).

The number of datasets supporting each relationship was quantified, and statistical significance was assessed using one-sided Fisher’s exact tests. To reduce bias arising from repeated experimental conditions or study-specific effects, phosphosite pairs were filtered based on:

1. Statistical significance (*p* < 0.05);
2. Minimum support across independent datasets;
3. Representation across multiple studies and conditions.

For each phosphosite pair, a co-regulation ratio was calculated as:

$$\mathrm{Co}\text{-}\mathrm{regulation}\;\mathrm{ratio}=\frac{\mathrm{Concordant}\;\mathrm{events}}{\mathrm{Discordant}\;\mathrm{events}}$$

Zero counts were replaced with one to avoid division errors.

### 4.5. Integration of Protein–Protein Interaction and Interactor Phosphosite Data

Protein–protein interactions and protein complex information were compiled from the Human Protein Reference Database (HPRD) [34], BioGRID [35], ConsensusPathDB (release 35) [36], CORUM [37], Biomolecular Interaction Network Database (BIND) [38], and RegPhos (version 2.0) [29]. For each kinase–metabolic enzyme pair, shared interactors were identified by intersecting kinase and enzyme interaction sets. Experimentally supported physical interactions were retained. Duplicate and homodimeric interactions were removed prior to downstream analyses. Interactor phosphosites were collected from experimentally validated events assembled in PTM databases and mapped to canonical UniProt sequences. Co-regulation analyses were subsequently performed for:

1. Metabolic enzyme phosphosite–interactor phosphosite pairs;
2. Kinase phosphosite–interactor phosphosite pairs.

These analyses provided an additional evidence layer for kinase prioritization.

### 4.6. Kinase Scoring and Prioritization Framework

To systematically prioritize biologically supported kinase–metabolic enzyme associations, we developed an empirical multi-layer scoring framework that integrates complementary evidence from kinase–substrate annotations, phosphoproteomic co-regulation, and protein–protein interaction networks. The framework was designed according to three guiding principles: (i) to prioritize experimentally supported kinase–substrate relationships as the primary source of evidence; (ii) to incorporate reproducible phosphoproteomic co-regulation while limiting the influence of highly redundant or extreme observations; and (iii) to include indirect interaction-derived evidence as complementary support without allowing it to dominate direct kinase–substrate evidence. Accordingly, four complementary evidence layers were integrated using progressively decreasing weights that reflect the relative confidence associated with each evidence layer. The framework is intended to prioritize candidate kinase–enzyme associations supported by multiple complementary evidence sources rather than predict novel kinase–substrate relationships. The mathematical formulation, variables, and biological rationale underlying each empirical scoring equation are described below.

#### 4.6.1. Scoring Components

For each metabolic enzyme phosphosite–kinase pair, four independent evidence layers were integrated.

##### Primary Interaction Score

Primary kinase–substrate evidence was assigned according to three confidence tiers corresponding to high-, medium-, and low-confidence evidence (3.25, 2.25, and 1.25, respectively), based on the level of experimental support reported across integrated kinase–substrate resources. High-confidence interactions represented experimentally validated kinase–substrate relationships supported by multiple orthogonal approaches, medium-confidence interactions comprised high-quality experimental or high-throughput evidence, and low-confidence interactions represented computational predictions or limited experimental evidence.

For kinase–substrate pairs reported by multiple databases, the primary interaction score was calculated as:

Primary Score=max(S)+w×(∑S−max(S))

where *S* represents evidence scores from individual databases, and *w* = 0.1, which assigns only a modest contribution from additional database annotations so that the highest-confidence evidence remains dominant while rewarding independent corroboration.

This formulation captures the strongest available evidence while providing a modest increase for independent corroboration across multiple resources. The weighted secondary contribution prevents redundant database annotations from artificially inflating the primary evidence score while rewarding agreement among independent data sources.

##### Kinase–Substrate Co-Regulation Score

For each kinase–substrate pair, phosphoproteomic co-regulation was quantified across independent quantitative phosphoproteomic datasets as:

$$\mathrm{Coreg}\;\mathrm{Score}=\alpha \times ln(1+r)\times (1+\beta \sqrt{n_{PMID}})$$

where *r* represents the effective co-regulation ratio calculated from concordant phosphorylation changes normalized by the number of kinase phosphosites, and $n_{PMID}$ denotes the number of independent phosphoproteomic studies supporting the association.

The logarithmic transformation compresses extremely large co-regulation ratios while preserving their relative ordering, thereby reducing the influence of outlier datasets. The square-root transformation of study count applies diminishing returns, allowing additional independent studies to increase confidence without disproportionately favoring highly studied phosphosite pairs. Parameters *α* = 0.5 and *β* = 0.2 were selected empirically, with α scaling the co-regulation score to a range comparable to the primary evidence and *β* providing diminishing returns for increasing numbers of supporting phosphoproteomic studies.

##### Kinase–Interactor Score

To incorporate indirect phosphoproteomic support through shared protein–protein interaction networks, kinase–interactor co-regulation was calculated as:

$$\mathrm{Interactor}\;\mathrm{Score}=\gamma \times \alpha \times ln(1+r_{KI})\times (1+\beta \sqrt{n_{PMID,KI}})$$

where $r_{KI}$ represents the effective kinase–interactor co-regulation ratio, and *γ* = 0.03.

Because this component represents indirect evidence derived from shared interaction networks rather than direct kinase–substrate relationships, *γ* = 0.03 was assigned to provide only modest contextual support without outweighing direct evidence.

##### Substrate–Interactor Score

Substrate–interactor co-regulation was similarly calculated as:

$$\mathrm{SubInt}\;\mathrm{Score}=\delta \times \alpha \times ln(1+r_{SI})\times (1+\beta \sqrt{n_{PMID,SI}})$$

where $r_{SI}$ represents the substrate–interactor co-regulation ratio, and *δ* = 0.01.

This component represents the most indirect evidence layer within the framework; therefore, *δ* = 0.01 was assigned to provide contextual support while exerting minimal influence on the overall prioritization.

#### 4.6.2. Final Score Aggregation

The final score for each kinase–substrate phosphosite pair was calculated as a weighted linear combination of the four evidence layers:

Final Score=0.4(Primary)+0.3(Coreg)+0.2(Interactor)+0.1(SubInt)

The weighting scheme assigns progressively decreasing contributions to the evidence layers according to their biological proximity to direct kinase–substrate relationships, with experimentally supported kinase–substrate evidence contributing most strongly, followed by direct phosphoproteomic co-regulation, kinase–interactor co-regulation, and substrate–interactor co-regulation. A weighted linear aggregation strategy was adopted because it provides transparent interpretation of individual evidence contributions while avoiding overfitting associated with more complex nonlinear integration methods. Parameter values were selected empirically based on iterative sensitivity analyses to preserve biological interpretability, prioritize direct experimental evidence, and maintain clear separation between evidence tiers.

Kinases were ranked independently for each phosphosite according to descending Final Score because individual phosphorylation sites often possess distinct candidate kinase repertoires. Independent phosphosite-specific ranking therefore enables direct comparison of candidate kinases competing for the same phosphorylation site rather than across unrelated phosphosites. Higher Final Scores indicate kinase–enzyme associations supported consistently across multiple independent evidence layers.

### 4.7. Analysis of Prioritized Kinase–Metabolic Enzyme Associations

#### 4.7.1. Identification of High-Confidence Kinase–Metabolic Enzyme Relationships

For downstream analyses, high-confidence kinase associations for each metabolic enzyme were defined using a rank threshold of ≤10 together with a phosphoproteomic support fraction of ≥15%, where the phosphoproteomic support fraction was calculated as:

$$\mathrm{Phospho}\;\mathrm{Fraction}=\frac{\mathrm{Coreg}\;\mathrm{Score}+\mathrm{Interactor}\;\mathrm{Score}+\mathrm{SubInt}\;\mathrm{Score}}{\mathrm{Final}\;\mathrm{Score}}$$

This metric estimates the proportion of the final score contributed by phosphoproteomic-derived evidence, enabling discrimination between kinase associations supported primarily by dynamic phosphoproteomic observations and those relying predominantly on database-derived annotations. Thresholds were selected following sensitivity analyses across multiple ranking and phosphoproteomic support combinations to balance confidence with pathway coverage for subsequent network analyses (Supplementary Table S6). Kinases were subsequently classified into priority tiers based on integrated evidence strength, phosphoproteomic support, and substrate coverage.

#### 4.7.2. Pathway-Level Regulatory Burden Analysis

Following kinase prioritization, each retained kinase association was defined as a unique kinase–metabolic enzyme–phosphosite relationship, with one kinase assigned independently to each phosphorylation site based on its integrated Final Score. Consequently, a single metabolic enzyme could be associated with multiple kinases across one or more phosphorylation sites. Unless otherwise specified, the term kinase association refers to these prioritized kinase–enzyme–phosphosite relationships.

For pathway-level analyses, kinase association density was calculated as the number of prioritized kinase–enzyme associations divided by the number of enzymes within each glucose metabolic pathway, thereby normalizing for differences in pathway size. Comparisons among pathways were performed using non-parametric statistical tests, and enrichment analyses were conducted to evaluate kinase association patterns across branch-point, flux-control, dual-control, and opposing enzyme categories.

#### 4.7.3. Pathway Preference Analysis

To compare kinase composition across pathways independently of pathway size, pathway preference analysis was performed for kinase groups and kinase families. Only kinase groups or families represented by at least 30 prioritized kinase–substrate associations were included. For each kinase family (or group), a Pathway Preference Score (PPS) was calculated as:

$$\mathrm{PPS}=\frac{N_{family,pathway}/N_{family,total}}{N_{\mathrm{pathway}}/N_{total}}$$

where *N_family,pathway_* is the number of prioritized kinase–substrate associations belonging to a given kinase family (or group) within a pathway, *N_family,total_* is the total number of prioritized associations assigned to that family (or group), *N_pathway_* is the total number of prioritized associations within the pathway, and *N_total_* is the total number of prioritized associations across all pathways. A PPS of 1 indicates representation equivalent to the global network background, values > 1 indicate relative over-representation, and values < 1 indicate relative under-representation.

#### 4.7.4. Enzyme-Level Kinase Association Analysis

To identify metabolic enzymes with the strongest overall kinase regulatory support, a composite enzyme kinase association score was calculated as:

$$\mathrm{Association}\;\mathrm{Density}\;\mathrm{Score}={log}_{2}\;\left(1+n_{kinases}\;\right)\times \bar{Final\;Score}\times \bar{Coregulation\;Score}\;$$

where *n_kinases_* represents the number of unique prioritized kinases associated with an enzyme. The logarithmic transformation reduces the influence of enzymes with exceptionally large numbers of kinase associations while preserving relative ranking among highly connected enzymes. Enzymes were ranked according to this score to identify candidate regulatory hubs within glucose metabolism.

### 4.8. Functional Classification of Metabolic Control Points

To enable systematic analysis of kinase association patterns across glucose metabolism, metabolic enzymes were classified according to their functional position within the curated glucose metabolic network. An operational classification framework was developed based on pathway topology and reaction directionality represented in KEGG [16], together with biochemical principles of metabolic control, and used consistently throughout subsequent analyses [58,59,77,78,79,80].

#### 4.8.1. Branch-Point Enzymes

Branch-point metabolites were defined as metabolites within the curated glucose metabolic network that are directly connected to two or more alternative metabolic fates represented in the analyzed pathways. The following metabolites were classified as branch metabolites:

- Glucose-6-phosphate;
- Fructose-6-phosphate;
- Glyceraldehyde-3-phosphate;
- Pyruvate;
- UDP-glucose.

Enzymes directly catalyzing reactions that consume or produce these metabolites were classified as branch-point enzymes.

#### 4.8.2. Flux-Control Enzymes

Flux-control enzymes were defined as enzymes catalyzing physiologically irreversible reactions within the curated glucose metabolic network based on KEGG reaction directionality. These enzymes were classified as flux-control enzymes.

#### 4.8.3. Dual-Control Enzymes

Enzymes satisfying both the branch-point and flux-control criteria were classified as dual-control enzymes, representing metabolic nodes that simultaneously occupy pathway branch points and irreversible reaction steps.

#### 4.8.4. Futile Cycle (Opposing) Enzymes

Opposing reaction pairs were identified from the forward and reverse reactions represented within the curated glucose metabolic network. Four opposing enzyme pairs were analyzed:

- Hexokinase (HK)/Glucose-6-phosphatase (G6PC);
- Phosphofructokinase (PFK)/Fructose-1,6-bisphosphatase (FBP);
- Pyruvate kinase (PK)/Phosphoenolpyruvate carboxykinase (PEPCK);
- Glycogen synthase (GYS)/Glycogen phosphorylase (PYG).

Forward and reverse enzymes were evaluated separately to compare shared and reaction-specific kinase associations.

### 4.9. Network Topology Analysis of Kinase–Enzyme Associations

A bipartite kinase–enzyme association network was constructed using high-confidence kinase–enzyme associations retained after prioritization (Rank ≤ 10 for each enzyme and phosphoproteomic support fraction ≥ 15%). Nodes represented protein kinases or glucose metabolic enzymes, whereas edges represented prioritized kinase–enzyme associations. Bipartite representation was adopted because kinase associations occur exclusively between protein kinases and metabolic enzymes, enabling direct characterization of network connectivity between the two node classes.

Network density together with kinase and enzyme degree distributions were calculated using NetworkX (version 3.3). Kinase degree was defined as the number of unique metabolic enzymes associated with each kinase, whereas enzyme degree represented the number of unique kinases associated with each metabolic enzyme.

Hub kinases were operationally defined as kinases with a degree exceeding twice the mean network degree, thereby identifying highly connected kinases substantially above average network connectivity while avoiding arbitrary percentile thresholds.

$$\mathrm{Hub}\;\mathrm{Threshold}=2\times \mu_{degree}$$

where $\mu_{degree}$ represents the mean kinase degree across the network. For each hub kinase, the numbers of branch-point, flux-control, dual-control, and opposing/futile-cycle enzyme targets were quantified together with the proportion of targets supported by shared protein–protein interaction evidence.

Kinase family annotations were obtained from KinBase [33] and UniProt classifications. To determine whether particular kinase families were overrepresented among hub kinases, hypergeometric enrichment analysis was performed by comparing the observed number of hub kinases within each family against the complete kinase population represented in the network. Resulting *p*-values were adjusted for multiple testing using the Benjamini–Hochberg false discovery rate (FDR), with FDR-adjusted *p* < 0.05 considered statistically significant.

### Degree-Preserving Null-Model Analysis

To evaluate whether the observed hub architecture exceeded random expectation, degree-preserving randomized bipartite networks were generated using the NetworkX (version 3.3) bipartite configuration model. This approach preserves the observed kinase and metabolic enzyme degree distributions while randomizing network connectivity. A total of 500 randomized networks were generated (random seed = 42), and self-loops introduced during randomization were removed before analysis.

For each randomized network, the maximum kinase degree was recorded and compared with that of the observed network.

Empirical *p*-values were calculated as:

$$p=\frac{N(\mathrm{Random}\;\mathrm{Max}\;\mathrm{Degree}\geq \mathrm{Observed}\;\mathrm{Max}\;\mathrm{Degree})}{500}$$

### 4.10. Tripartite Network Construction and Identification of Bridge Proteins

A tripartite kinase–interactor–enzyme network was constructed using shared interacting proteins identified during kinase prioritization to investigate indirect network connectivity between the kinase and metabolic enzyme layers. Bridge proteins were defined as proteins reported to interact with both kinase and metabolic enzyme layers within the tripartite network. Nodes represented kinases, interacting proteins, or metabolic enzymes, whereas edges represented kinase–interactor or interactor–enzyme relationships.

For each bridge protein, two connectivity measures were calculated:

$$K_{deg}=\mathrm{Number}\;\mathrm{of}\;\mathrm{connected}\;\mathrm{kinases}$$

$$E_{deg}=\mathrm{Number}\;\mathrm{of}\;\mathrm{connected}\;\mathrm{enzymes}$$

A bridge score was calculated as:

$$\mathrm{Bridge}\;\mathrm{Score}=K_{deg}\times E_{deg}$$

thereby prioritizing proteins that simultaneously connected multiple kinases and multiple metabolic enzymes rather than proteins predominantly connected to only one network layer.

To additionally account for global network position, betweenness centrality was calculated using NetworkX, and a combined ranking score was computed as:

Combined Score=Bridge Score×Betweenness Centrality

Bridge proteins with bridge scores above the 90th percentile were considered high-confidence bridge candidates and were ranked according to the combined score for downstream analyses and network visualization.

### 4.11. Statistical Analysis and Software Environment

All data processing, statistical analyses and visualization were performed in Python (version 3.12.3) using pandas (2.1.3), NumPy (1.26.2), SciPy (1.13.0), NetworkX (3.3), matplotlib (3.8.4), and seaborn (0.13.2). Network topology analyses, including degree, network density, betweenness centrality, and graph connectivity calculations, were performed using NetworkX.

Continuous variables were summarized using descriptive statistics, including means, medians, standard deviations, interquartile ranges, and frequency distributions, as appropriate. Group comparisons involving non-normally distributed network variables were performed using Mann–Whitney U tests. Effect sizes (r) were calculated for pairwise comparisons. Hypergeometric tests were used to assess enrichment of kinase families and metabolic control-point categories relative to the complete kinase population represented in the analyzed network. Multiple-testing correction was performed using the Benjamini–Hochberg false discovery rate (FDR) procedure where applicable. Statistical significance was defined as *p* < 0.05 or FDR-adjusted *p* < 0.05, as appropriate.

### 4.12. Data Availability

All scripts used for kinase scoring and downstream analyses performed in this study are publicly available through the GitHub repository (v1.0.0): https://github.com/elizabethsusmi/kinase-metabolic-network (accessed on 25 July 2026).

## 5. Conclusions

We developed a multi-layer prioritization framework that integrates kinase–substrate annotations, phosphoproteomic co-regulation, and protein–protein interaction evidence to systematically identify high-confidence kinase–metabolic enzyme associations across glucose metabolism. By integrating multiple complementary evidence layers, this framework provides a systematic approach for prioritizing biologically supported kinase–enzyme associations beyond individual data sources. Using this framework, we show that kinase associations preferentially converge at metabolic control points, including branch-point and flux-controlling enzymes, rather than being uniformly distributed across the network. Network analyses further identified a small subset of hub kinases coordinating large portions of the glucose metabolic network and candidate bridge proteins linking the kinase and metabolic enzyme layers. Collectively, these findings provide a systems-level view of kinase–metabolic enzyme associations across glucose metabolism and establish a resource for prioritizing candidate kinase–enzyme relationships for future experimental investigation.

## Figures and Tables

**Figure 1:**
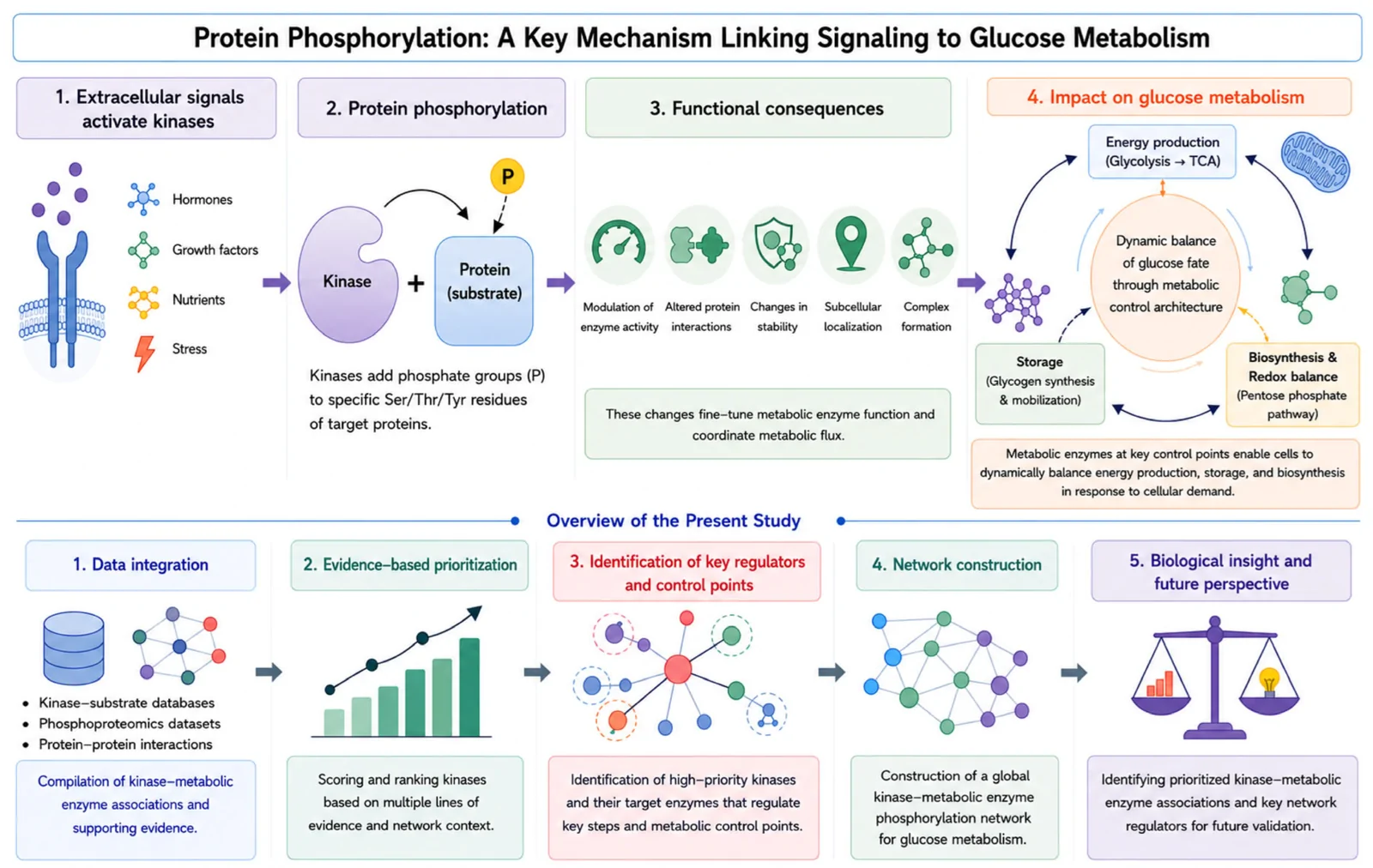
**Conceptual overview of protein phosphorylation in glucose metabolism and the study workflow.** The upper panel illustrates how extracellular signals activate protein kinases that phosphorylate metabolic enzymes, influencing their activity, interactions, stability, localization, and complex formation to regulate glucose metabolism. The lower panel summarizes the computational framework developed in this study, including data integration, evidence-based kinase prioritization, network analysis, and identification of prioritized kinase–metabolic enzyme associations for future experimental validation.

**Figure 2:**
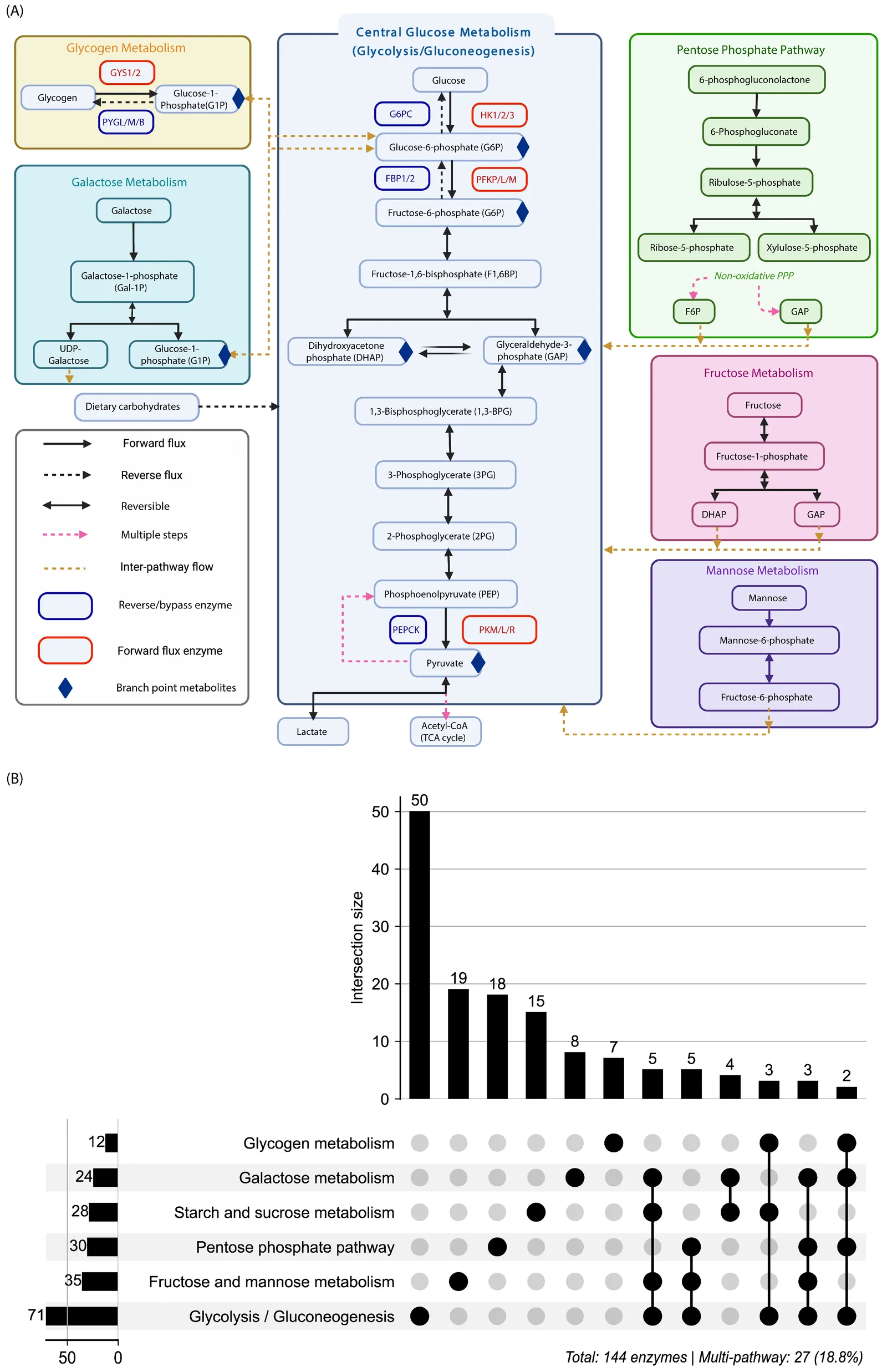
**Overview of the glucose metabolic network and pathways overlap analysis.** (**A**) Schematic representation of the glucose metabolic network analyzed in this study, comprising core glucose utilization pathways together with associated carbohydrate conversion and dietary carbohydrate entry pathways, including glycolysis/gluconeogenesis, glycogen metabolism, the pentose phosphate pathway, galactose metabolism, fructose metabolism, and mannose metabolism. Major branch-point metabolites, irreversible flux-control steps, futile-cycle pairs, and representative enzyme families are highlighted to illustrate pathway interconnectivity and key metabolic control points involved in glucose utilization, storage, and biosynthetic branching. Solid arrows indicate irreversible forward reactions, double-headed arrows indicate reversible reactions, and dashed arrows represent gluconeogenic bypass or multi-step reactions. (**B**) Distribution and overlap of glucose metabolic enzymes across pathways. UpSet plot representing the distribution and overlap of metabolic enzymes across six glucose metabolism pathways: glycolysis/gluconeogenesis, fructose and mannose metabolism, the pentose phosphate pathway, starch and sucrose metabolism, galactose metabolism, and glycogen metabolism. Horizontal bars indicate the total number of enzymes per pathway, while vertical bars represent the size of each pathway intersection. Filled circles in the matrix denote pathway membership for each intersection. In total, 144 enzymes were analyzed, including 27 enzymes present in multiple pathways.

**Figure 3:**
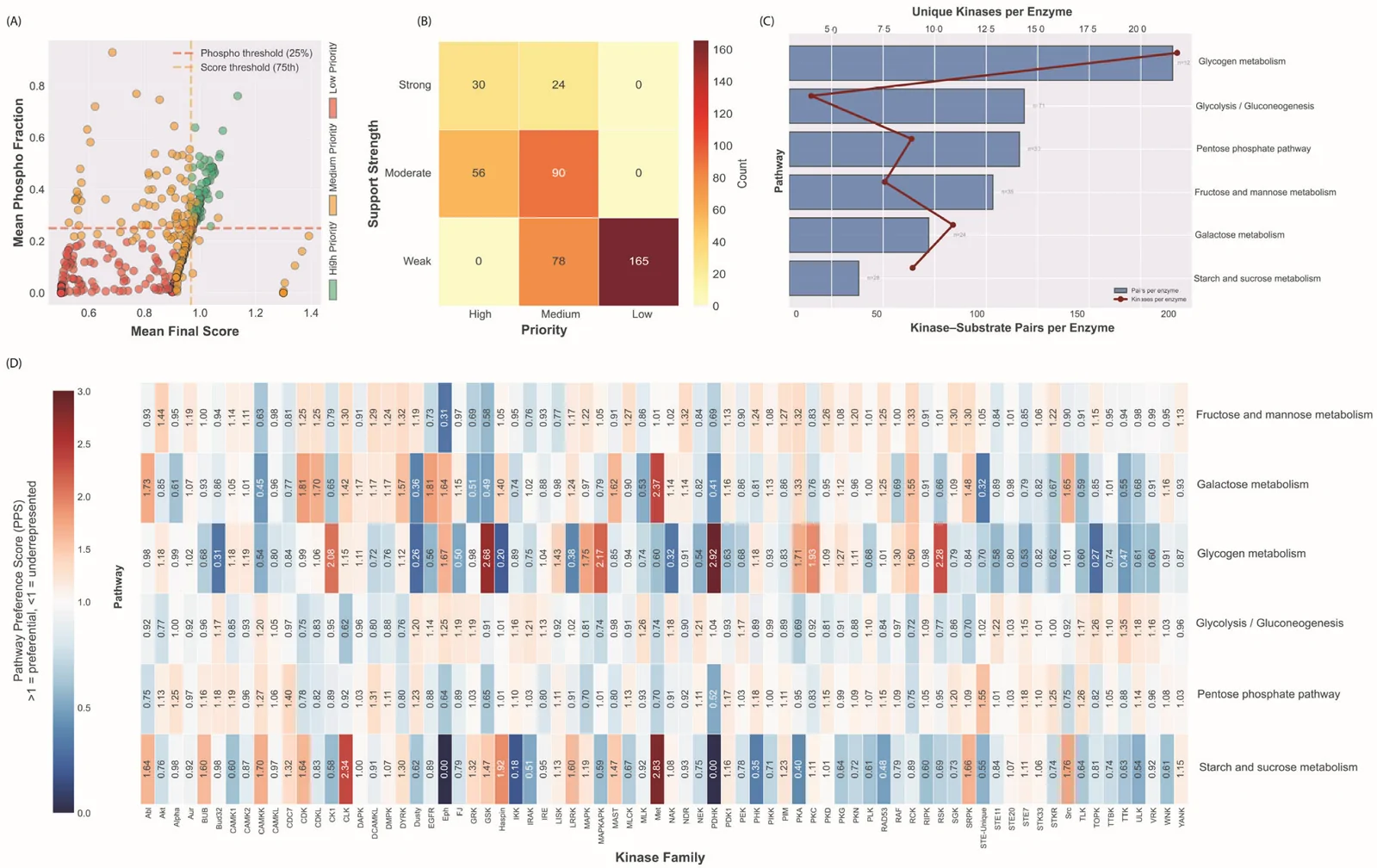
**Prioritization and pathway distribution of phosphoproteomic-supported kinase associations across glucose metabolism** (**A**) Scatter plot showing kinase prioritization based on mean final score and mean phosphoproteomic support fraction. Each point represents an individual kinase retained in the prioritization framework. Kinases are colored according to priority classification (High, Medium, Low). Dashed lines indicate the phosphoproteomic support threshold (25%) and the score threshold corresponding to the 75th percentile of final scores. Kinases with high phosphoproteomic support and elevated final scores cluster in the upper-right region of the plot, whereas lower-priority kinases are concentrated at lower phosphoproteomic support values. (**B**) Heatmap summarizing the distribution of kinases across combined priority and phosphoproteomic support categories. Rows represent phosphoproteomic support strength classifications (Strong, Moderate, Weak), and columns represent kinase priority groups (High, Medium, Low). Cell values indicate the number of kinases within each category combination. Most low-priority kinases exhibited weak phosphoproteomic support, whereas high-priority kinases were predominantly associated with moderate-to-strong phosphoproteomic support. (**C**) Pathway-level kinase association density across glucose metabolic pathways. Horizontal bars represent the number of kinase–substrate associations normalized per enzyme for each pathway, while the overlaid line indicates the number of unique kinases per enzyme. Glycogen metabolism showed the highest kinase association density and the largest number of unique kinases per enzyme, whereas starch and sucrose metabolism exhibited the lowest kinase association density among the analyzed pathways. (**D**) Heatmap showing pathway preference scores (PPS) for kinase families across glucose metabolic pathways. PPS values represent normalized observed/expected association ratios, where values > 1 indicate higher relative representation within a pathway compared to the global network background and values < 1 indicate lower relative representation. The color scale ranges from blue (lower relative representation) to red (higher relative representation). Glycogen metabolism showed relatively higher representation of AGC-, CK1-, and Atypical-associated kinase families, whereas galactose metabolism and starch and sucrose metabolism displayed comparatively higher representation of several tyrosine kinase-associated families. Most kinase families exhibited PPS values close to the global background distribution across pathways.

**Figure 4:**
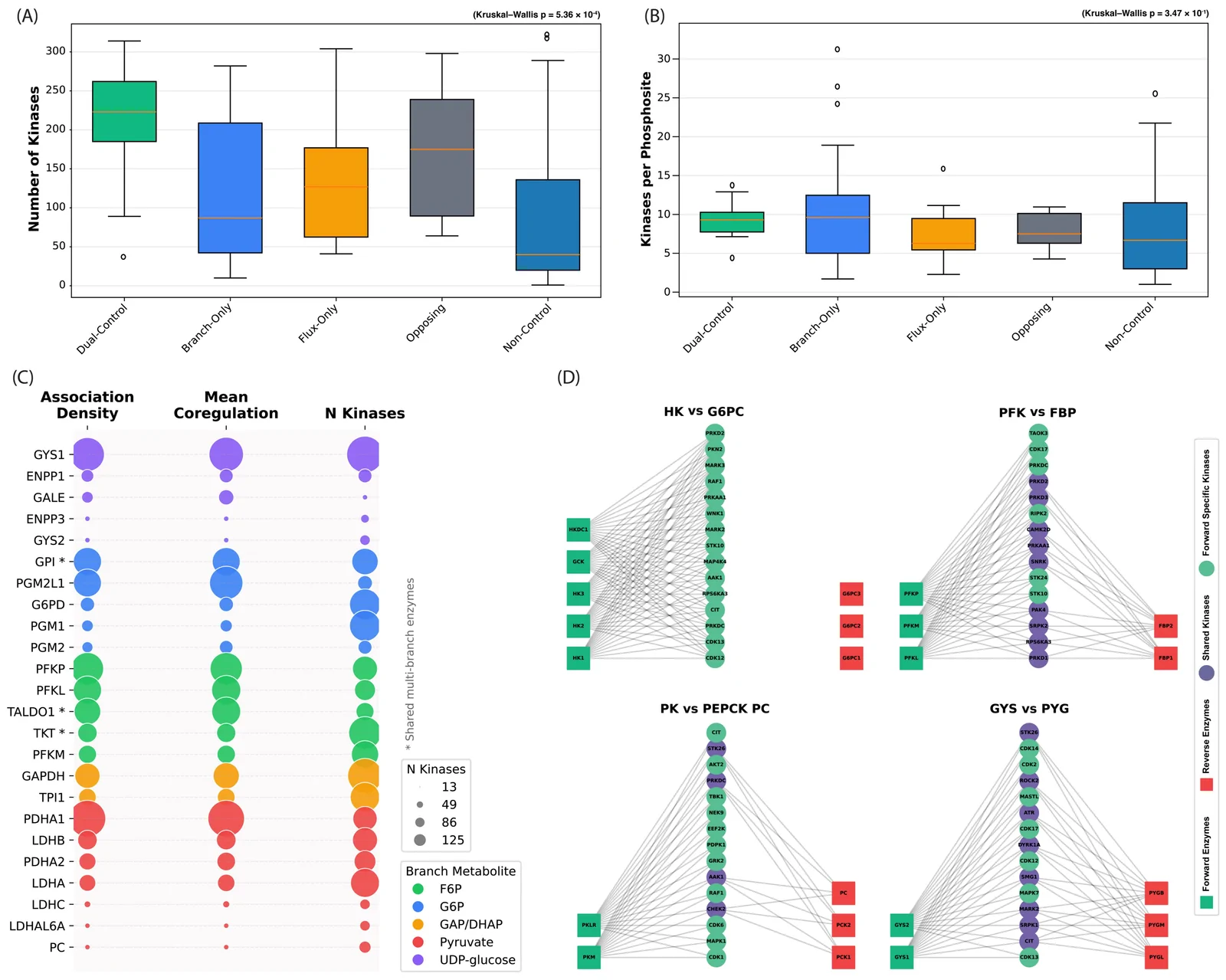
**Distribution of prioritized kinase associations across metabolic control-point enzyme categories.** (**A**) Boxplot showing the total number of prioritized kinase associations per enzyme across dual-control, branch-only, flux-only, and non-control enzyme categories using high-confidence kinase–substrate associations (Rank ≤ 10; phosphoproteomic fraction ≥ 15%). Statistical significance across categories was evaluated using the Kruskal–Wallis test. (**B**) Boxplot showing phosphosite-normalized kinase associations (number of associated kinases per phosphosite) across enzyme categories. Boxplots display the median, interquartile range, and outliers. (**C**) Bubble heatmap summarizing kinase association patterns across major branch-point enzymes. Bubble size reflects the relative magnitude of each metric within its respective column, and bubble color indicates branch-metabolite grouping. Columns show the association density score, mean phosphoproteomic co-regulation support, and the numbers of prioritized high- and medium-confidence kinases. (**D**) Network representation of prioritized kinase associations across four opposing metabolic reaction pairs (HK/GCK–G6PC, PFK–FBP, PK–PEPCK/, and GYS–PYG). Edge connections represent prioritized high-confidence kinase–enzyme associations.

**Figure 5:**
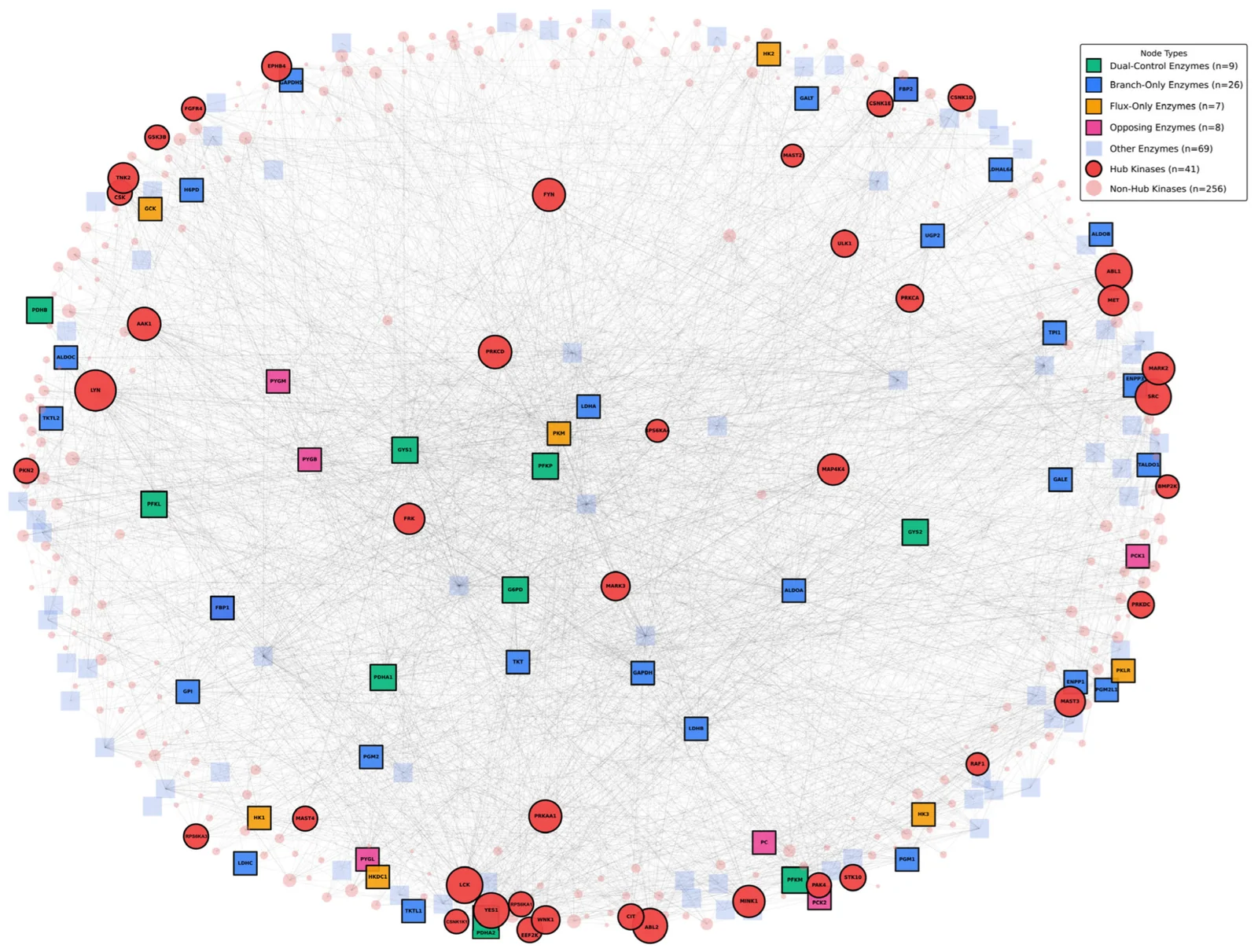
**Bipartite network architecture of kinase association patterns across glucose metabolism.** Global bipartite kinase–enzyme association network across glucose metabolism. The network comprises 297 protein kinases, including 41 hub kinases, connected to 117 glucose metabolic enzymes through prioritized high-confidence kinase–enzyme associations derived from the integrated scoring framework. Circular nodes represent kinases, with node size proportional to network degree (number of associated metabolic enzymes). Square nodes represent glucose metabolic enzymes and are color-coded according to their metabolic control architecture (dual-control, branch-control, flux-control, opposing/futile-cycle, and other metabolic enzymes). Gray edges denote prioritized kinase–enzyme associations. The network illustrates the global organization of prioritized kinase–enzyme associations across glucose metabolism and the distribution of hub kinases relative to metabolic control-point enzymes.

## Data Availability

All processed data supporting the findings of this study are provided within the article and the accompanying Supplementary Information. The original curated phosphoproteomic datasets used for the analyses are available from the corresponding author upon reasonable request.

## Supplementary Materials

The following supporting information can be downloaded at https://www.mdpi.com/article/10.3390/ijms27219402/s1.
