# Supplementary material for: Kinase Regulation Through Metabolic Control Points Directs Glucose Fate
Source: Int J Mol Sci. 2026 Oct 22;27(21):9402. doi: 10.3390/ijms27219402 (PMC13762879; doi:10.3390/ijms27219402)
Supplement: Supplementary file 1 [file ijms-27-09402-s001.zip › Supplementary Figures.pdf]

Supplementary Figures

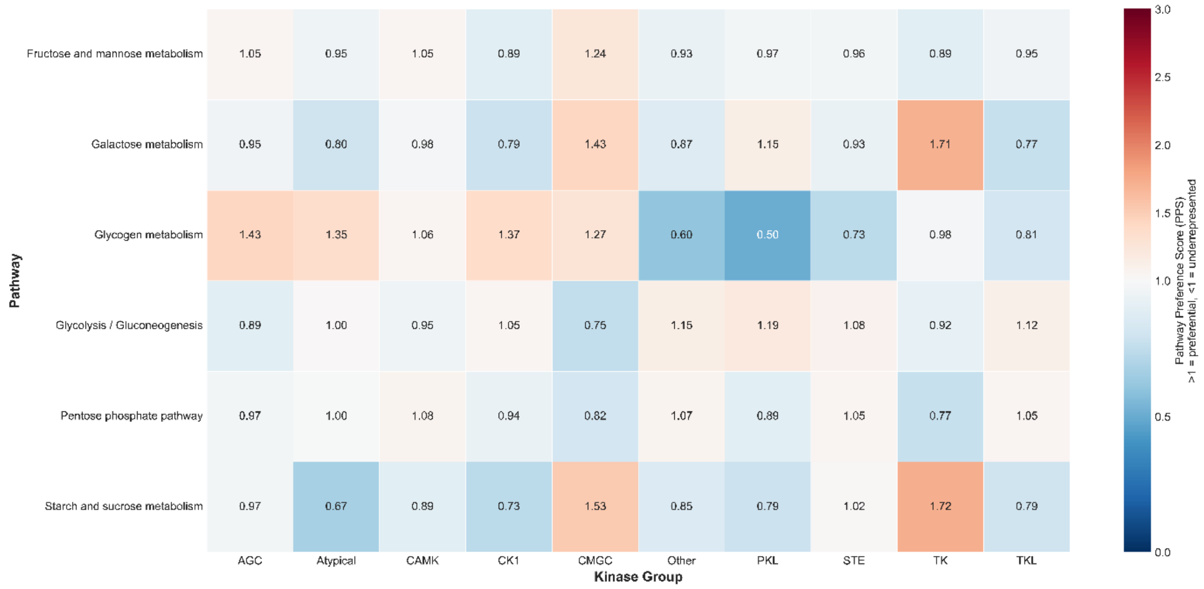

Figure S1. Pathway preference analysis of kinase groups across glucose metabolic pathways.

Normalized pathway preference scores (PPS) were calculated for each kinase group by comparing the observed number of kinase–enzyme associations within each glucose metabolic pathway to the expected number based on the global kinase-group distribution across the complete glucose metabolic network. A PPS value of 1 (white) indicates representation equal to the global background, values >1 (red) indicate pathway-specific enrichment, and values <1 (blue) indicate relative under-representation. Numbers within each cell represent the corresponding PPS values. This normalization corrects for differences in pathway size and overall kinase-group abundance, enabling comparison of kinase-group preferences across glycolysis/gluconeogenesis, glycogen metabolism, pentose phosphate pathway, galactose metabolism, fructose and mannose metabolism, and starch and sucrose metabolism.

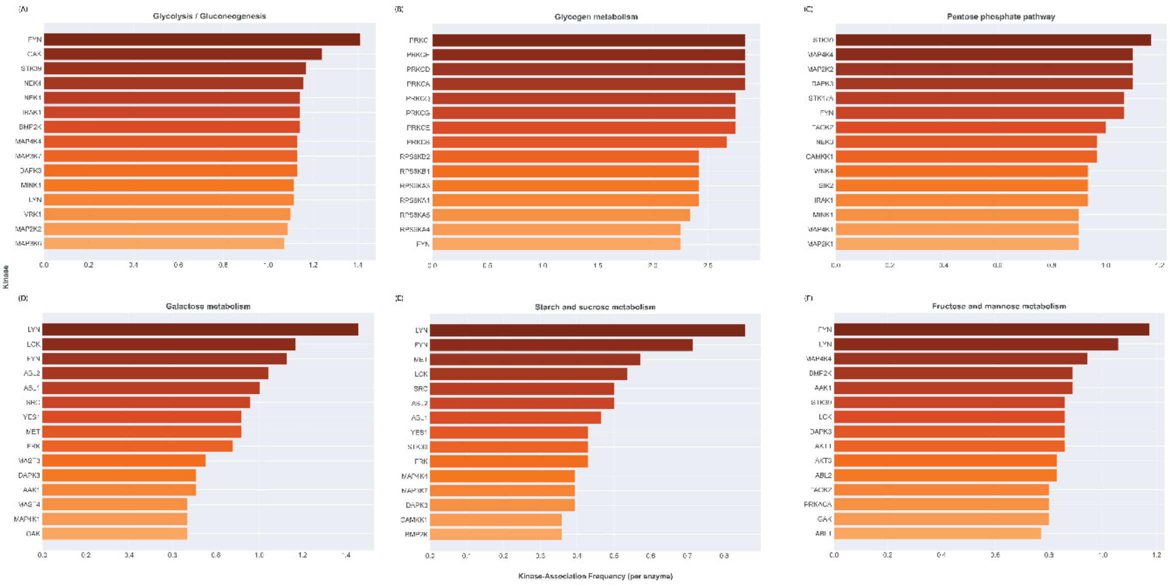

Figure S2. Top kinases associated with individual glucose metabolic pathways.

Top-ranked kinases for each glucose metabolic pathway based on kinase-association frequency normalized by the number of enzymes within the pathway. Panels show (A)

glycolysis/gluconeogenesis, (B) glycogen metabolism, (C) pentose phosphate pathway, (D) galactose metabolism, (E) starch and sucrose metabolism, and (F) fructose and mannose metabolism. The x-axis represents the normalized kinase-association frequency (mean number of kinase associations per enzyme within each pathway), while the y-axis lists the corresponding kinases. This analysis identifies kinases exhibiting preferential association with individual metabolic pathways while accounting for differences in pathway size.

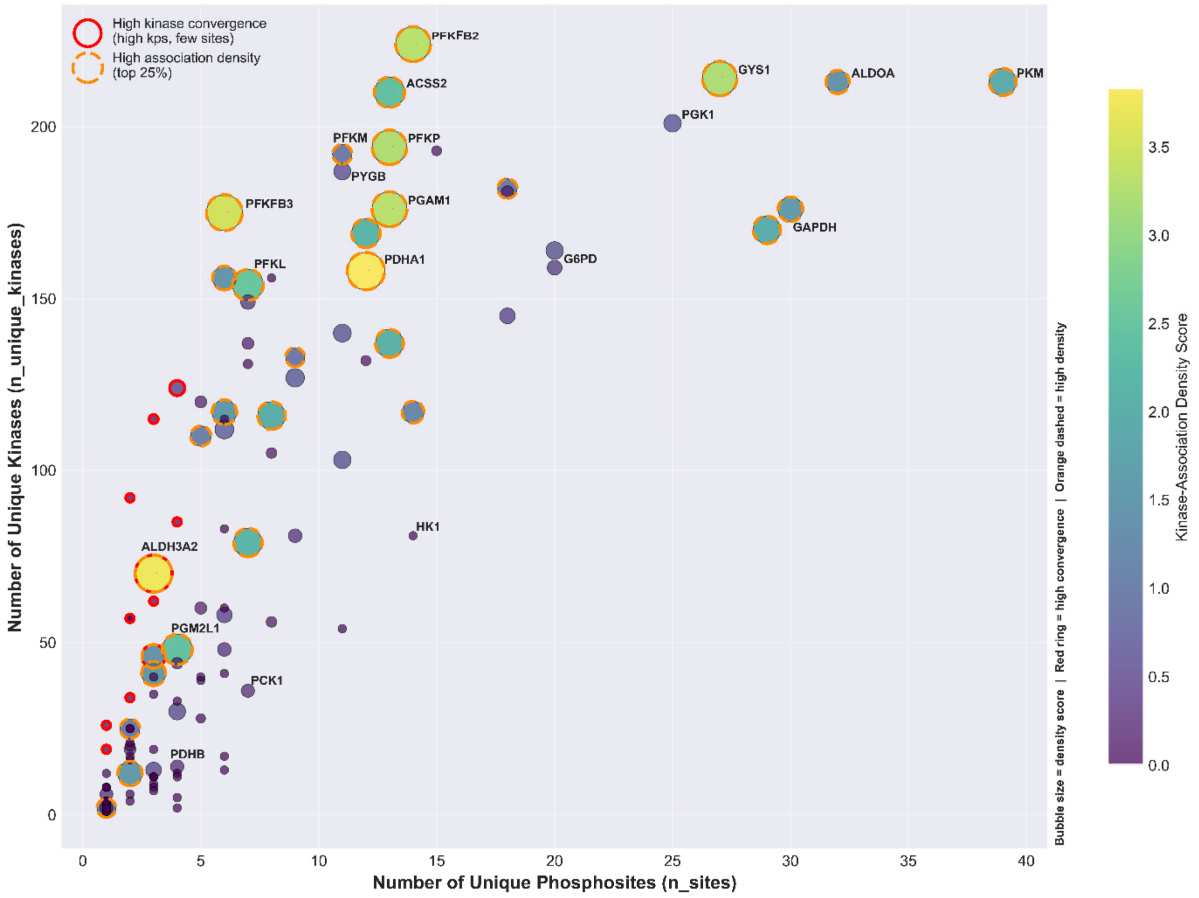

Figure S3. Enzyme-level kinase-association landscape across glucose metabolism.

Scatter plot showing the distribution of glucose metabolic enzymes according to the number of unique phosphorylation sites (x-axis) and the number of unique associated kinases (y-axis). Bubble size and color represent the composite kinase-association density score, integrating kinase-association breadth, association confidence, and phosphoproteomic co-regulation support. Enzymes outlined with a red solid circle exhibit high kinase convergence, indicating numerous kinase associations concentrated on relatively few phosphosites, whereas enzymes enclosed by an orange dashed circle represent the top 25% of enzymes ranked by kinase-association density score. Selected enzymes with the highest kinase-association density or convergence are labeled to highlight prominent regulatory nodes within the glucose metabolic network.

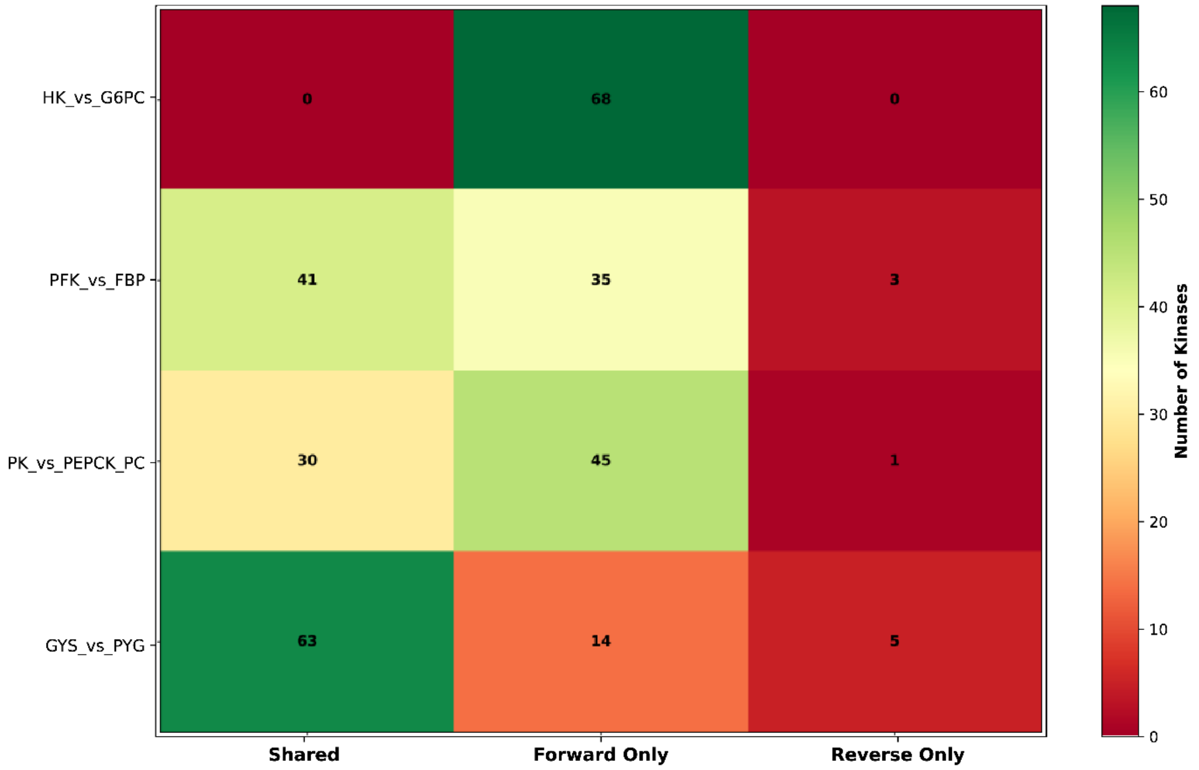

Figure S4. Quantitative summary of shared and distinct kinase associations across opposing glucose metabolic reaction pairs.

Heatmap summarizing the number of prioritized kinase associations shared between forward and reverse enzyme groups across four opposing metabolic reaction pairs within glucose metabolism. Rows correspond to opposing reaction pairs, and columns indicate shared kinases, forward-associated kinases, and reverse-associated kinases. Cell values indicate the number of kinases within each category. The HK/GCK–G6PC pair showed complete separation of kinase associations, whereas the GYS–PYG pair exhibited the largest shared kinase-association component. Intermediate overlap patterns were observed for the PFK–FBP and PK–PEPCK/PC pairs. Color intensity corresponds to the number of associated kinases.

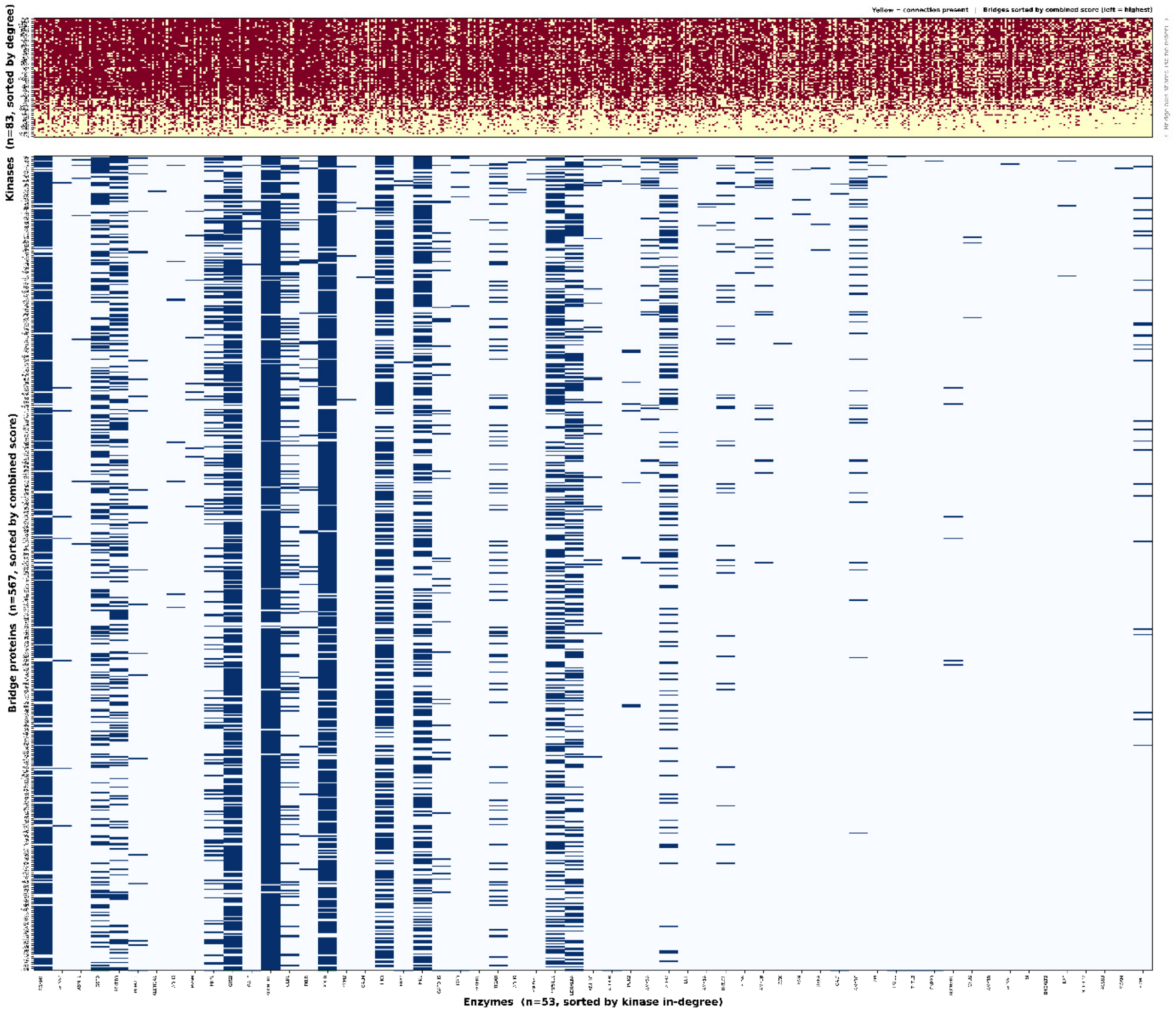

Figure S5: Tripartite kinase–bridge protein–enzyme interaction network visualized as paired heatmaps. The upper heatmap depicts kinase–bridge protein interactions, where rows correspond to kinases ranked by network degree and columns correspond to bridge proteins ranked by combined bridge score. The lower heatmap displays bridge protein–enzyme interactions using the same bridge protein ordering, with rows representing bridge proteins and columns representing glucose metabolic enzymes ranked by kinase in-degree. Colored cells indicate the presence of experimentally supported kinase–bridge or bridge–enzyme interactions. Sharing the bridge protein axis across both panels enables visualization of putative indirect regulatory paths from kinases to metabolic enzymes through bridge proteins. The figure illustrates the hierarchical organization of kinase signaling and identifies highly connected bridge proteins linking kinase and metabolic enzyme layers within the tripartite network.
